# News Items

**Published:** 1872-03-01

**Authors:** 


### News Items.

The two celebrated museums collected by Ruysch were sold, the first in 1717, to Peter the Great, for 30,000 florins, and sent to St. Petersburg; the second was sold after his death, in 1731, to either the King of Poland or the Elector of Saxony, for 20,000 florins. Portions of the second museum have been examined and found to be worthless. The first, however, which still forms a part of the Anatomical Museum of the Imperial Academy of Sciences, is in a good state of preservation.—*Phil. Med. Times.*

Small-pox still prevails in this city and vicinity, and since our last report the number of victims has much increased, averaging over 80 for each of the past two weeks. Philadelphia reports the enormous average of 200 deaths weekly.—*New York Med. Record*, *Feb.* 1, 1872.

The Importation of Opium.—The money value of opium imported through the Custom House of New York City, in the year 1871, was $1,299,091.—*Ibid.*

Small-pox in England.—In the year 1870, 1,259 deaths were registered in the seventeen prominent cities and towns in England. No less than 13,174 persons fell victims to the disease, in the same towns, in the year 1871. The highest small-pox mortality in London, during the 31 years, 1840–1870, was in 1863, when 2,012 fatal cases were reported. Last year 7,876 fatal cases occurred in London alone; of which 2,400 occurred in the first, 3,241 in the second, 1,255 in the third, and 980 in the fourth quarters. The proportion of fatal cases to the population of the seventeen towns was 18 per 10,000; the ratio in London was 24, in Norwich 30, in Liverpool 39, in Wolverhampton 41, in Newcastle-on-Tyne 54, and in Sunderland 86.—*Ibid.*

The Jerusalem German Hospital.—During the last two years the German deaconesses, in their hospital at Jerusalem, have cared for 1,414 patients, belonging to nine religious sects. Out of this large number only 46 have died.—*Ibid.*

The Hop Pillow.—During the illness of the Prince of Wales, when all other remedies failed to produce a soporific effect, the old-fashioned hop pillow was placed under his head and quiet sleep followed.—*Ibid.*

The American Mother Growing Less Prolific.—Dr. Toner, of Washington, D. C., the statistician, in his diagram, shows that what is held up as the peculiar shame of Massachusetts is equally true of all her sister States. It is shown that only one-half as many children are now born to each thousand women as in 1800, and that there has been a regular decrease from one decade of years to another.—*Ibid.*

